# Continental evolution influenced by relamination of deeply subducted continental crust

**DOI:** 10.1038/s41561-026-01963-w

**Published:** 2026-05-05

**Authors:** Daniel Gómez-Frutos, Antonio Castro, Attila Balázs, Taras Gerya

**Affiliations:** 1 Museo Nacional de Ciencias Naturales (MNCN), Consejo Superior de Investigaciones Científicas (CSIC), Madrid, Spain; 2 Department of Earth and Planetary Sciences, ETH Zurich, Zurich, Switzerland; 3 Present Address: Institute of the Earth and Environment, University of Portsmouth, Portsmouth, UK

**Keywords:** Geodynamics, Geochemistry, Precambrian geology, Petrology, Tectonics

## Abstract

Post-collisional magmatism has played a critical role in modifying the continental crust since the Archaean, 3 billion years ago. The dual mantle–crust signature of calc-alkaline post-collisional magmas requires a hybrid source involving both reservoirs, the formation of which is not well understood. Here we use an integrated thermomechanical and magmatic experimental approach to constrain the geodynamic causes of mantle–crust hybridization and post-collisional magmatism, which reveals their association with subduction of continental lithosphere. Our numerical models predict pervasive relamination of deeply subducted continental crust onto the base of the overriding plate, owing to the decoupling of the buoyant silica-rich crust from the subducting continental lithosphere. In this setting, post-collisional magmatism is sourced from a hybrid domain following efficient mechanical mixing between the relaminated crust and mantle peridotite. Our laboratory melting experiments demonstrate that the magmatic products from this hybrid source reproduce the natural compositional trend of post-collisional igneous rocks. This mechanical–chemical interaction between relaminated crust and mantle lithosphere is recorded in magmatic isotopic signatures throughout Earth’s history and, given the similarity between Phanerozoic post-collisional calc-alkaline rocks and Archaean sanukitoids, implies that crust–mantle hybridization has occurred since Precambrian plate tectonics.

## Abstract (web summary)

Post-collisional calc-alkaline magmatism has originated from the mixing and hybridization of relaminated continental crust and mantle peridotite since the Archaean, according to numerical simulations of continental subduction and melting experiments.

## Main

The origin of the continental crust in Earth ultimately responds to igneous processes, where magma is segregated from the mantle and incorporated into the crust. Understanding how the continents originated demands addressing the challenges around post-collisional calc-alkaline (Caledonian I-type) magmatism, featured in collisional orogens as large silicic intrusions emplaced during the post-collisional stage of an orogeny. A key paradox prevails among the origin of post-collisional calc-alkaline magmatism: natural rocks display a hybrid crust–mantle geochemical fingerprint that points to both a mantle^1–3^ and crustal^4–6^ origin. This hybrid signature is also featured in the mafic–intermediate (that is, sanukitoid) suite^7,8^, a metasomatized mantle-sourced magmatic series^9,10^ formed during episodes of lithosphere extension and closely related to post-collisional granites since the Archaean^1,11,12^. The involvement of a metasomatized mantle source in generating post-collisional magmas is thus widely accepted. In contrast, the mechanism responsible for mantle metasomatism is still under debate. Although recent geochemical inference points to fluids derived from the subducted continents^13–17^, fluids appear unable to transport the required geochemical budget^18–20^. As a result, the cause-and-effect relationship between continental collision, mantle metasomatism and post-collisional magmatism remains unclear.

Continental collision can be extensively studied by thermomechanical modelling. Coupled modelling evidence has granted basic insights into the styles of subduction and collision^21–23^ and a better understanding of the crustal architecture of fold and thrust belts^24,25^ and processes such as delamination^26,27^ or slab break-off^28,29^. In this context, common hybridization mechanisms on the mantle lithosphere include the gradual hydration from the subducting oceanic plate^30,31^, sediment plume interactions from the subduction interface^22^ or molten diapirs from the subducted mélange^32,33^. Nevertheless, most of this variability refers to the oceanic stage of subduction and arc magmatism^34^. In contrast, post-collisional magmatism is predominantly depicted by decompression melting of asthenospheric windows^27,30^. Whereas partial melting of a hydrated asthenosphere translates into ultra-mafic to mafic intrusions, natural orogens display a clear predominance of silicic rocks, and basaltic magmas are unable to reproduce the natural compositions through magmatic differentiation^35^. Together with peel-back delamination, which may be responsible for the destruction and replacement of the mantle lithosphere^27^, whether mantle metasomatism and magmatism are coeval remains unknown. Regardless, the consistent and globally common geochemistry of post-collisional magmatism^1^ implies that the involved mechanisms operate universally. Notably, mafic–intermediate (that is, sanukitoid) magmas have consistently appeared in collisional orogens since the late Archaean^36,37^, suggesting that the mechanisms driving post-collisional magmatism were operative during the primordial stages of the continental crust^10^.

Here we present an integrated approach using thermomechanical numerical modelling and experimental petrology to establish the mechanism for mantle metasomatism and post-collisional magmatism. Numerical models are designed to analyse the geodynamic conditions for mantle metasomatism during continental collision, pointing to crust–mantle solid-state mixing after crustal relamination. Complementary high-pressure melting experiments are used to explore the melting behaviour of mechanically mixed crust–mantle assemblages. By relamination, we refer to the decoupling of a positively buoyant subducted felsic crust from the denser mafic crust and its rise and incorporation into the lithosphere of the overriding plate^38,39^. This framework allows for assessing the influence of deep crustal relamination in crustal evolution, consistent with the long-term occurrence of post-collisional magmatism since the Archaean.

## Controls on crustal relamination

**Fig. 1:**
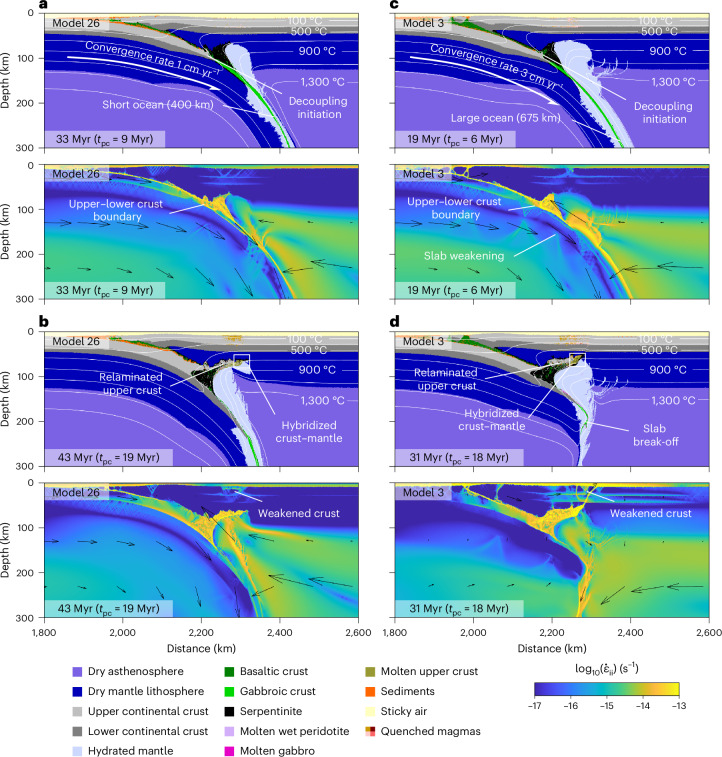
Dynamics of continental subduction and deep crustal relamination. Representative numerical models of deep crustal relamination, plotted for lithology and deformation intensity (second strain invariant, ${\dot{\epsilon}}_{\rm{ii}}$). Black arrows represent the velocity vector field. Timestamps in each panel indicate total model duration and, in parentheses, the t ime elapsed since continental collision (*t*_pc_). **a**, Model 26 after collision and relamination initiation. **b**, Model 26 during underplating of the relaminant in the overlying lithosphere. **c**, Model 3 after collision and relamination initiation. **d**, Model 3 after slab break-off, featuring relamination.

**Extended Data Fig. 1:**
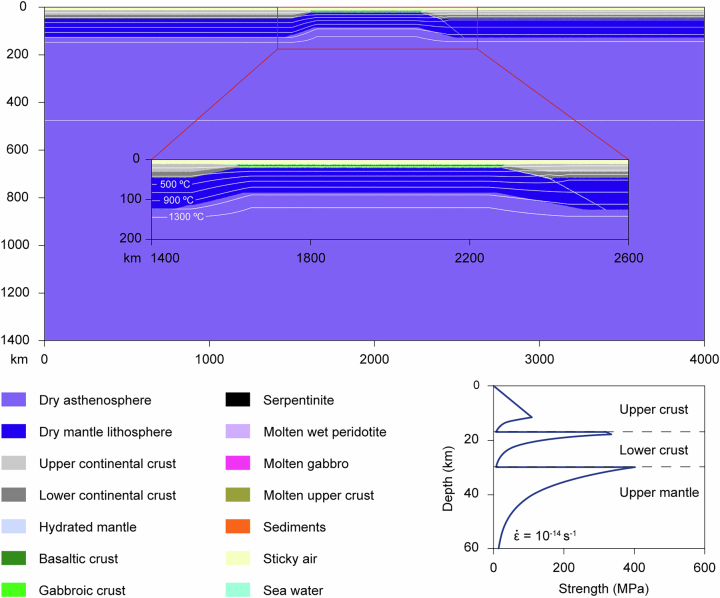
Starting numerical setup. Starting numerical setup for the numerical experiments and rheological profile of the lithosphere.

Our modelling of continental collision shows the predominance of continental subduction and crustal relamination after collisional orogeny (results reported in Supplementary Table 1). Numerical models share a common evolution during the oceanic subduction stage, where the overlying lithosphere undergoes gradual hydration from the subducting ocean up to ocean closure. Figure 1 illustrates the typical collision evolution in our models both in the presence and absence of slab break-off. Despite a gradual decrease of about 40% in the initial subduction velocities (Methods provide boundary conditions), strong upper mantle rheology and sufficient slab pull allow for continued continental subduction, and the eventual arrival of the leading edge of the slab to the transition zone initiates a new episode of trench retreat. During this stage, progressive strain accumulation in the subducted upper–lower crust interface causes the buoyancy force from the upper crust to overcome the resistance between the upper and lower crust, initiating decoupling and relamination at a consistent depth of about 100 km (Fig. 1a,c). Following the gradual entrainment of relaminant into the overlying lithosphere and faulting of the overlying plate, the buoyancy of the continental mass evens out with the peridotite and becomes stalled (Fig. 1b,d). Whereas trench retreat may peel back some of the relaminated crust into the asthenosphere, a substantial volume remains in the mantle lithosphere throughout the whole orogenic process. Lastly, weakening in the overriding plate results in relaminant exhumation at the later stages of the models^39^. The decoupling between the crustal layers is primarily driven by the low viscosity of the lowermost part of the lighter upper crust (Extended Data Fig. 1). Whereas a rheologically stronger upper crust leads to no relamination and coupled behaviour of the continental crust^22,30^, petrological and seismological constraints point towards a layered continental crust since the Archaean^9,38^, with a cumulate-like lower crust and a more felsic upper crust^34^. Such inference supports our stratified crustal models by suggesting that a non-uniform layered rheology is the most common scenario in nature.

**Extended Data Fig. 2:**
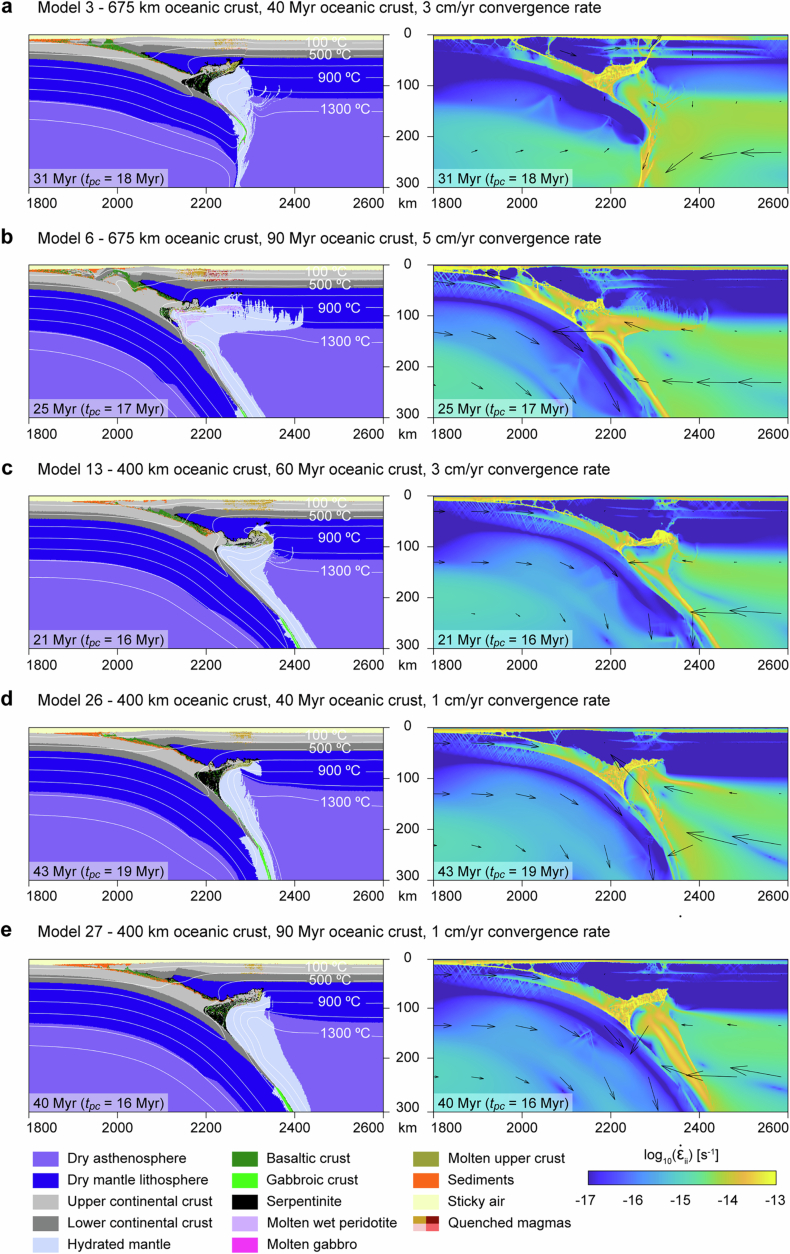
Parameter sensitivity study. **a–e**, Representative models from the parameter sensitivity study.

**Extended Data Fig. 3:**
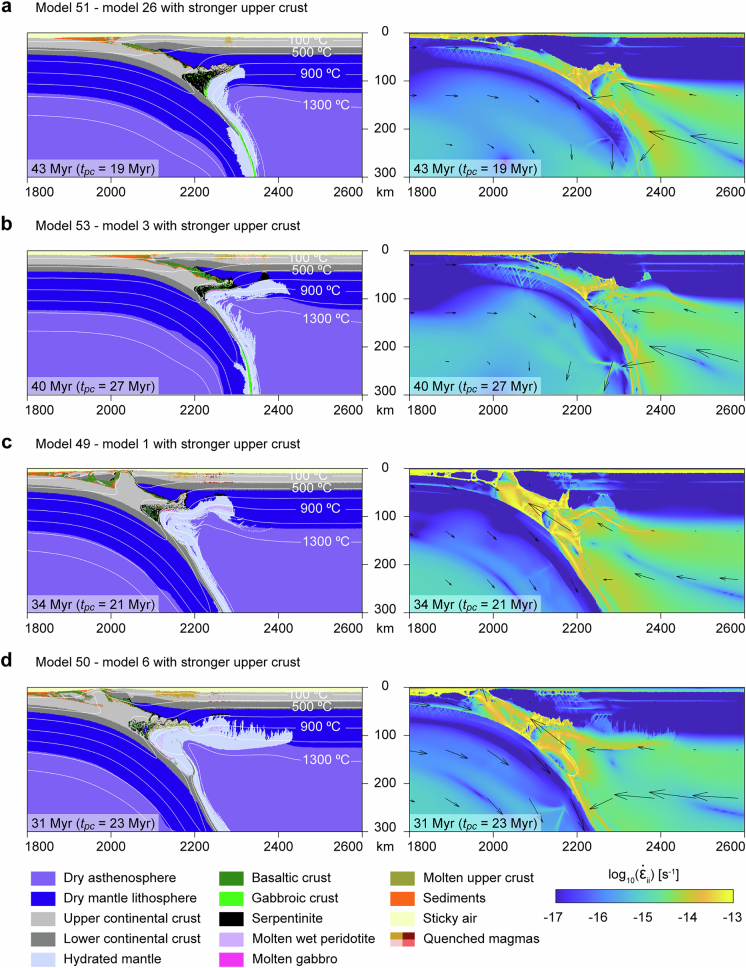
Models with stronger upper crust. **a–d**, Results of the reference models with stronger upper continental crust, sin(φ) = 0.6-0.3, plotted for lithology and deformation intensity.

**Extended Data Fig. 4:**
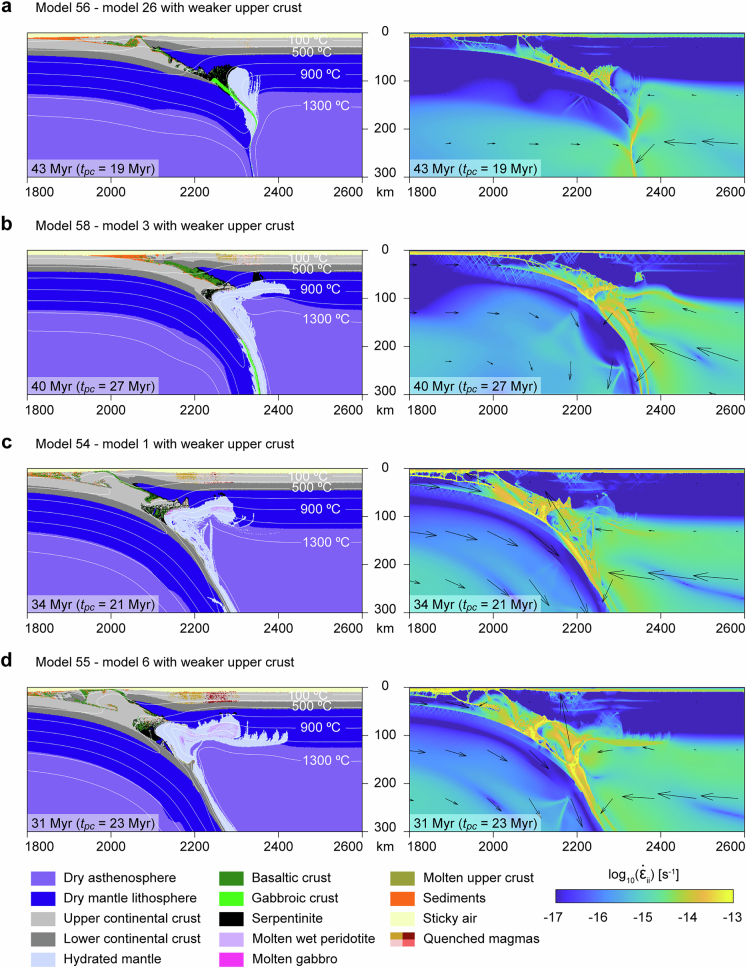
Models with weaker upper crust. **a–d**, Results of the reference models with weaker upper continental crust, sin(φ) = 0.15-0.1, plotted for lithology and deformation intensity.

**Extended Data Fig. 5:**
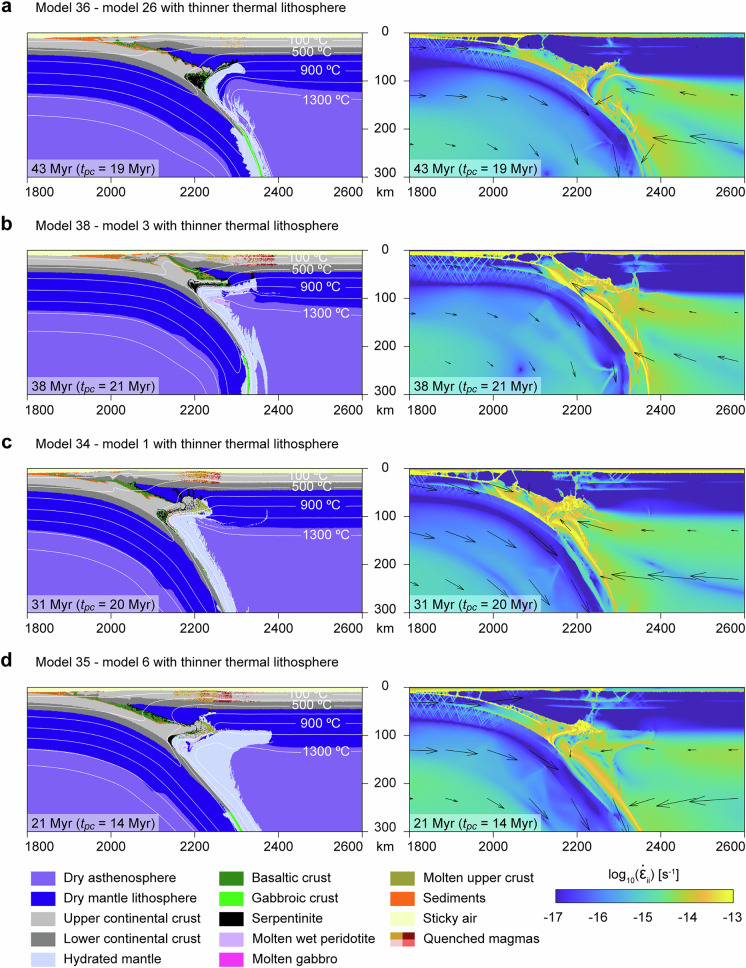
Models with thinner thermal lithosphere. **a–d**, Results of the reference models with a hotter, 8.62 K/km thermal gradient in the upper plate, plotted for lithology and deformation intensity.

Model sensitivity study on convergence velocities, length and age of the oceanic plate shows that our proposed relamination scenario is robust, occasionally affected by parameter variability. Compared to previous collision models^22,24,39^, implementation of brittle–ductile damage and grain size reduction^40^ is essential and leads to intense mechanical crust–mantle mixing and to slab break-off during the trench retreat stage (Fig. 1c,d). Although convergence velocities mostly affect the orogen architecture^25^, high convergence rates induce higher strain in the slab that, together with a younger ocean, result in slab break-off. Slab break-off from the lower crust notably decreases the negative buoyancy of the subducting continent, causing a rebound to shallower depths and slowing convection in the mantle wedge. Although early slab break-off may prevent the subducted crust from reaching sufficient depth for detachment of the upper crust (Extended Data Fig. 4b), relamination otherwise remains a persistent process throughout the investigated model parameter space. Larger oceanic plates develop larger thermal anomalies in the mantle wedge (Extended Data Fig. 2) and higher hydration from the subducting ocean, ensuring appropriate conditions for partial melting. Further parameter tests on different upper crustal rheologies and a thinner thermal lithosphere show that the occurrence of relamination remains consistent (Extended Data Figs. 3–5). A much weaker upper crust prevents relamination in two of the four tested models due to the occurrence of slab break-off before relamination initiation (compare Fig. 1 with Extended Data Fig. 4a–b), also inducing a higher trench migration velocity compared to cases with stronger rheologies^41^. None of the models report perturbations on the overlying lower crust prone to trigger magmatism.

Our models conducted at modern-day mantle potential temperatures indicate that relamination is primarily controlled by the rheological structure of the lithosphere. Under Archaean conditions, ultra-hot orogens feature relaminated blocks of buoyant, felsic continental crust^42^, potentially producing a behaviour similar to that in our models by underplating the lithospheric mantle^43–45^. Higher mantle temperatures during the Archaean may increase slab break-off frequency^46^. However, our models indicate that this does not necessarily prevent relamination (Fig. 1d), with the critical factor remaining the subducting continent reaching sufficient depth to initiate buoyant decoupling of the upper crust. Although collisional models under Archaean conditions remain a goal for future work, these considerations suggest that relamination was mechanically plausible during the Archaean.

## Interaction between relaminated crust and mantle

**Fig. 2:**
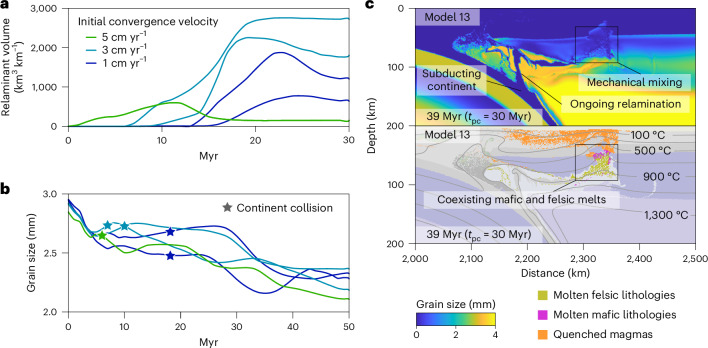
Volume of relaminated crust and grain size evolution and associated melting conditions in the reference models. **a**, Volume evolution of the relaminated crust in an approximately 50,000 km^2^ window in the overlying lithosphere in the selected models from the parameter study (5 cm yr^−1^ model 6, 3 cm yr^−1^ models 3 and 13, 1 cm yr^−1^ models 26 and 27; Extended Data Fig. 2). **b**, Grain size evolution in the same window and reference models. Smoothing was applied using a moving average with a ± 1 Myr window. **c**, Model yielding the most brittle–ductile damage in the lithosphere among the reference models (full model evolution is available in Supplementary Video 1). The two panels represent grain size (top) and a lithology plot with molten lithologies highlighted (bottom, colour map as in Fig. 1). Timestamps indicate total model duration and, in parentheses, the time elapsed since continental collision (*t*_pc_).

Relamination rates (that is, relaminated crust volume over time) remain steady throughout the tested parameters. The relaminant volume in the lithosphere peaks ca. 16 ± 5 Myr after collision, with higher convergence velocity inducing earlier relamination (Fig. 2a). At this stage, the overlying lithosphere is deformed at high strain rates, with widespread faulting creating a favourable scenario for magma extraction (Figs. 1b,d and 2b). Notably, the delay in the peak volume of relaminated crust is consistent with the magmatic hiatus observed in natural orogens, where magmatism typically follows collision after a variable lag of several million years (refs. ^47,48^), probably reflecting differences in convergence velocity among colliding plates (Fig. 2a). Contrarily, relamination does not control the brittle–ductile damage of the lithosphere^40^. The grain size of the overlying lithosphere displays a steady decline during oceanic subduction and a faster decrease during continental subduction and collision (Fig. 2b). Relaminant volumes show a positive correlation with grain size reduction in the overlying lithosphere, ensuring an efficient interaction between the relaminant and the surrounding peridotite (Fig. 2c).

**Extended Data Fig. 6:**
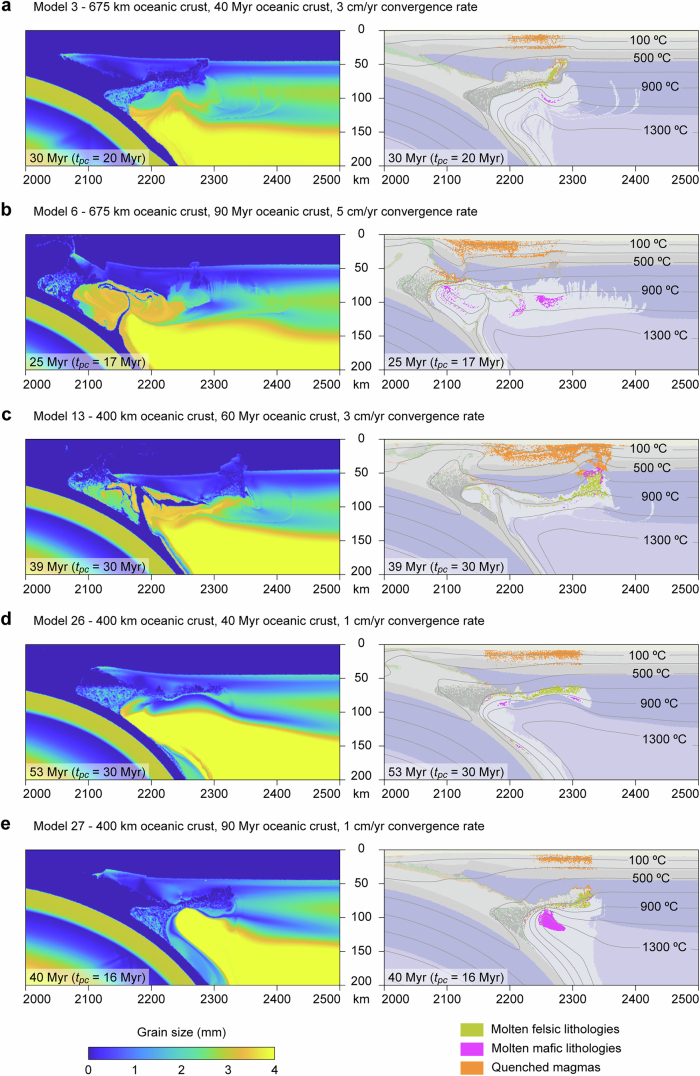
Grain size and melting in the reference models. **a–e**, Grain-size and highlighted melting regions in the reference models.

Expected three-dimensional heterogeneity along the upper–lower crust boundary suggests that relamination occurs as localised instabilities in the subducted continent drip feeding the overlying lithosphere. Furthermore, oblique subduction geometries can influence the along-trench variation of oceanic and continental subduction^49,50^, causing variable buoyancy and resistance forces that also influence upper plate deformation and melting conditions. The combination of these factors can result in collision and magmatism being diachronous in case a sufficient thermal anomaly does not exist in the lithosphere during crustal relamination. In contrast, thinner thermal lithosphere ensures more efficient relamination due to the higher buoyant force from the upper crust and larger thermal anomalies above 900 °C (Extended Data Fig. 6). Combined decompression and isotherm ascent (Fig. 2c) ensure near-solidus melting, producing coexisting felsic and mafic melts (Fig. 2c and Extended Data Fig. 6). Rather than magma mixing, we interpret this co-occurrence as a result of our melting algorithm treating each lithology independently (Methods), with crust–mantle hybridization occurring in solid state. Importantly, the presence of mafic melts confirms that conditions exceed the solidus of mafic lithologies, a process likely amplified under higher Archaean mantle temperatures. Further Moho temperature anomalies can be facilitated by arc magmatism after prolonged subduction of larger oceanic plates. Onto the magmatic output, the arrival of crustal blocks into the peridotite results in a hybrid lithospheric mantle, where the metasomatic event required for post-collisional magmatism can be explained by deep crustal relamination.

## Deep hybrid source for post-collisional magmatism

Interaction between a felsic relaminated crust and peridotite can yield pervasive metasomatism in the lithosphere, with progressive heating during orogenic evolution potentially melting the resulting silicic reservoir. Following this inference, our numerical modelling-inspired melting laboratory experiments test the products from a hybrid source using variable proportions of peridotite mixed with an upper crustal contaminant, mirroring the mechanical, solid-state mixing predicted by our models (Methods). The synthesized products comprise an intermediate liquid in equilibrium with a solid assemblage dominated by orthopyroxene, clinopyroxene and olivine (Supplementary Table 2).

**Fig. 3:**
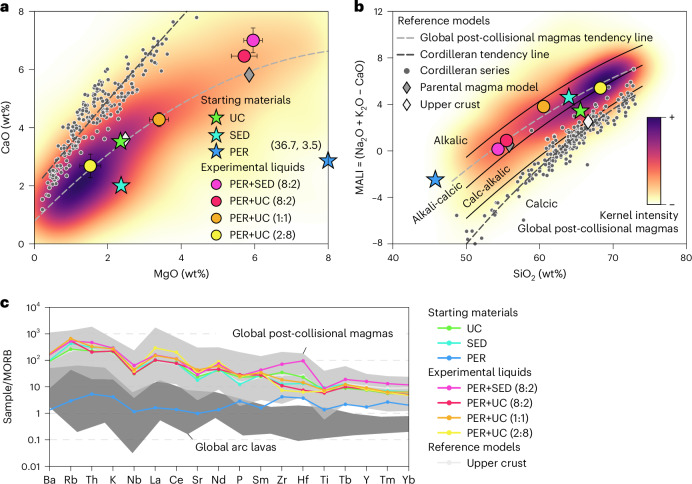
Geochemistry of high-pressure experimental products and comparison with post-collisional and cordilleran magmatism. **a**, Major-element composition of experimentally synthesized products in the CaO–MgO projection. Starting materials and runs are labelled UC (upper crust), SED (sediment) and PER (peridotite); full sample and run names can be found in Supplementary Table 2. The coloured background shows a kernel-density representation of a global compilation of post-collisional magmas (comparison with individual world-class batholiths in Extended Data Fig. 7). Reference models are the average composition of the upper continental crust^60^, a parental model calculated from 2,569 analyses of mafic–intermediate post-collisional magmas^36^ (SiO_2_ 48–63 wt%) and the Peninsular Ranges batholith representing the Cordilleran series. Error bars represent ±1*σ* of the analysis from the experimental results and are shown only when larger than the marker size. **b**, Major element composition of the experimentally synthesized products in the MALI (Na_2_O + K_2_O − CaO) projection, using the same samples and reference fields as in **a**. **c**, Normalized trace-element composition of experimental melts. Reference models are a global compilation of post-collisional magmas and a global compilation of Cordilleran magmas from arc volcanoes^61^. Data are available in Supplementary Table 2. All comparative data can be found in Supplementary Table 3.

**Extended Data Fig. 7:**
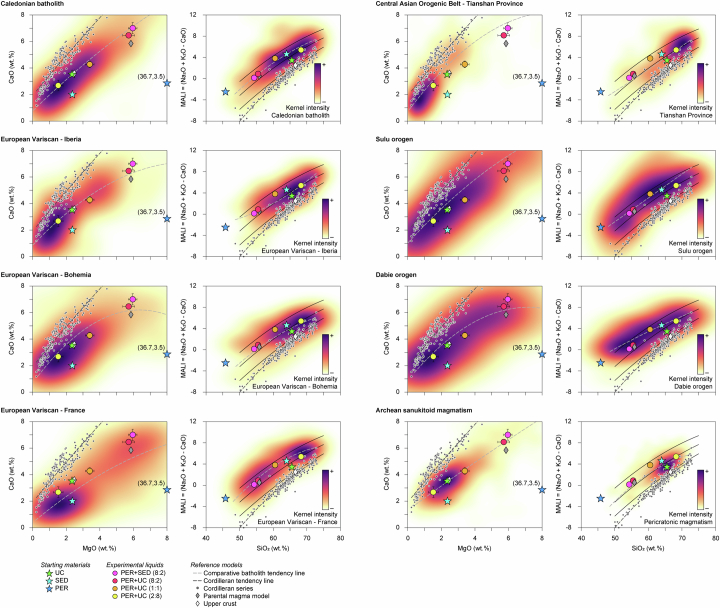
Geochemical comparison with global post-collisional magmas. Geochemical comparison between synthesized experimental liquids and post-collisional magmas worldwide.

Our new experiments demonstrate that the reaction between a hybrid peridotite-relaminant source produces liquids with post-collisional fingerprint (that is, sanukitoid; Fig. 3). Despite the heterogeneous nature of post-collisional rocks, this geochemical imprint consisting of high K_2_O, MgO, large-ion lithophile elements (LILE) and light rare earth elements (LREE) and low CaO is featured globally^12^, and distinguishes them from the other nominal subduction magmatic suite, namely Cordilleran magmatism (Fig. 3 and Extended Data Fig. 7). Experimental melts from runs with a dominant peridotite fraction fall at the head of the post-collisional array, matching the average composition of the parental mafic–intermediate magmas. Runs using increasing crustal proportions produce granodioritic liquids akin to post-collisional granites. All synthesized liquids manifest enrichment in MgO (Fig. 3a), supplied by reaction with the abundant Mg-rich phases in the peridotite. This highlights the necessity of mantle intervention in the reaction and thus excludes a crustal-only process. They also showcase a consistent depletion in CaO, a prominent feature of post-collisional magmas likely resulting from comparatively lower CaO contents in the continent relaminant compared to the oceanic input in arc settings^34,51,52^. The experimental liquids are notably enriched in alkalis (Fig. 3b), particularly in K_2_O. The run using a sediment contaminant yielded melts with higher K_2_O and lower Na_2_O contents compared to using the average upper crust, resembling the composition of the also ubiquitous ultrapotassic intrusions^15,53^. Additionally, all experimental liquids showcase enrichment in trace elements (notably in LILE and LREE) relative to the starting mixtures that overlap the post-collisional magmas field, also distinct from the Cordilleran geochemical fingerprint (Fig. 3c).

Synthesized products exhibit a notably low modal proportion of melt at 1,200 °C, highlighting the role of grain-to-grain melt migration in hybrid mantle sources. In this sense, a damaged lithosphere due to isostatic perturbations after relamination and ensuing post-collisional extension (Figs. 1 and 2) is a favourable setting for magma segregation and extraction at very low partial melting degrees. Altogether, these considerations suggest that the compositional range of post-collisional magmatic suites can be reproduced through heterogeneities within the relaminated crust, magmatic fractionation and variable degrees of partial melting, where the melt composition is buffered by the presence of peridotite in the starting compounds. The initial magmatic products from this crust–mantle hybrid source behave like cotectic systems^54^, whereas potential deviations of the natural rocks from the experimentally determined cotectic trends can be ascribed to accumulation and liquid segregation during ascension and emplacement^55^. Moreover, such a hybrid source is only sensitive enough to detect variations between hybridization with oceanic or continental crust, in which hybridization with the former results in I-type Cordilleran magmas^52,56,57^, while according to our results, the latter produces I-type post-collisional magmas.

## Crust–mantle hybridization since the Archaean

**Fig. 4:**
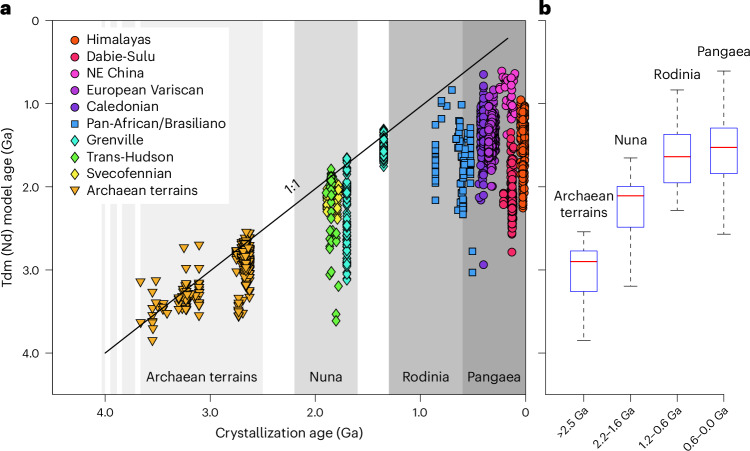
Evolution of the Nd model ages throughout the history of the Earth. **a**, Evolution of Nd model ages of post-collisional magmatism in the most studied orogens in the world. Grey bars represent each supercontinent cycle^62^. The 1:1 line describes the evolution of an unmodified, progressively more depleted mantle. **b**, Boxplot data distribution for post-collisional magmas within each supercontinent cycle, representing the statistical median (red line) and interquartile range (Q1–Q3); whiskers extend to the most extreme data points within 1.5 times the interquartile range, with outliers excluded. Data compilation entails 1,519 whole rock isotope analyses from post-collisional magmas and is available in Supplementary Table 3.

Our combined numerical and experimental evidence shows that, owing to the mechanical–chemical interaction between the relaminating deeply subducted upper continental crust and the overlying orogenic lithosphere, post-collisional magmas are predominantly sourced in a hybridized mantle lithosphere. The proposed genesis of lithospheric hybrid sources provides a self-consistent explanation for the occurrence of post-collisional magmatism after continental collision and reconciles its dual mantle–crust signatures, a feature that is directly testable using post-collisional magmatic products. Our new global-scale geochemical assessment suggests that the long-term continued interaction between subducted continental crust and lithospheric mantle explains the consistent radiogenic Nd enrichment relative to the depleted mantle evolutionary stage (Fig. 4a), with the isotopic signature inherited from the source. Because small proportions of relaminant substantially modify the isotopic signature of the magmatic products^3^, variability among different intrusions in the same orogen likely corresponds to the different nature of the relaminated crust in the hybrid mantle–crustal source. Remarkably, the featured geochemistry of each batholith is correlated with the composition of the pre-collisional continental crust of the subducting plate. Notable enrichment in Nd isotopes is registered in settings where the subducting crust features an Archaean terrain, such as the India–Eurasia collision in the Himalayas (*T*_DM_ = 1,590 ± 350 Ma) or the Yangtze craton subducting below the Sino-Korean craton in the Sulu-Dabie orogen (*T*_DM_ = 2,186 ± 210 Ma); whereas the presence of a younger pre-collisional continental crust results in more modest Nd enrichment, as registered in the Variscan orogen (*T*_DM_ = 1,450 ± 160 Ma) and the Caledonian orogen (*T*_DM_ = 1,370 ± 270 Ma) in Europe, where crustal relamination has been documented^58,59^. This observation is supported by the negative correlation between *T*_DM_ ranges and crystallization ages (Fig. 4b), caused by an evolving, gradually more heterogeneous continental crust to be hybridized in the mantle through relamination in each subsequent supercontinent cycle.

In the specific case of Archaean cratons, the presence of sanukitoid magmas is particularly enlightening. Their resemblance to Phanerozoic post-collisional magmas^12^ implies that continental crust–mantle interaction is essential for their generation. The consistently enriched nature of post-collisional magmas throughout Earth’s history demands mantle hybridization with a felsic crustal material. Given the buoyancy of felsic crust, such material is most plausibly introduced into the mantle via subduction rather than by crustal dripping. This indicates that deep, hybrid crust–mantle sources, probably driven by continental subduction and crustal relamination, were active in Precambrian plate tectonics. Accordingly, future thermomechanical models must incorporate hybrid melting to realistically simulate post-collisional magmatism. More broadly, our results identify post-collisional magmatism as a key tracer for the fate of subducted continents, showing how deep crustal reworking has played a major role in continental evolution since the Archaean.

## Methods

### Model set-up

Numerical experiments were carried out with the thermomechanical two-dimensional code I2VIS^63,64^, which solves the governing equations of conservation of mass, momentum and energy on a staggered Eulerian grid. Lagrangian markers are used to carry material properties, temperature and water contents. They are initially distributed randomly throughout the Eulerian grid, and displacement at each time step is calculated using the computed velocity field. Viscoplastic non-Newtonian rheologies and variable thermal conductivity are used in all numerical experiments^65–70^ (Supplementary Table 1), accounting for adiabatic, radiogenic, frictional and latent heating sources. Numerical C codes used for the modelling are available in Zenodo^71^.

The non-uniform rectangular grid consists of 1,361 × 351 nodal points and uses a high-resolution scheme of 0.5 km. The computational domain covers a two-dimensional field 4,000 km wide by 1,400 km deep. All boundaries are free slip. The upper free-surface boundary condition is implemented by using a 12-km-thick ‘sticky’ air/water layer^72^ with low density (1 kg m^−3^ above 9 km, 1,000 kg m^−3^ below 9 km) and viscosity (10^17^ Pa s). The continental crust has a total thickness of 35 km. It is subdivided into 20 km of upper continental crust with an average felsic composition and 15 km of lower continental crust with an average mafic composition. The upper crust has a wet quartzite weak rheology and the lower crust has a plagioclase strong rheology^73^. The underlying lithospheric mantle is 90 km thick and uses a dry olivine rheology, same as the asthenosphere. The oceanic domain comprises an ocean plate with a variable length of 675 km or 400 km and equally variable thermal age of 90 Ma, 60 Ma and 40 Ma (Supplementary Table 1). The oceanic crust consists of 3 km of basalts over a 5 km gabbro layer for a total of 8 km. The thickness of the oceanic lithosphere is calculated as a function of the chosen initial age (homogeneous for the whole oceanic plate) and an oceanic geotherm, resulting in an oceanic lithosphere of simplified thermal structure. The thermal structure of the initial set-up is defined by a laterally uniform cooling and respective geotherm^67^ with an upper boundary with constant temperature of 273 K, a Moho temperature of 933 K in the overriding plate, a thermal gradient of 6.57 K km^−1^ until a temperature of 1,603 K and then an overall quasi-adiabatic mantle geotherm of 0.5 K km^−1^. No heat flux exists across the vertical boundaries. The models used a gravitational acceleration of 9.81 m s^−2^. The physical properties of the lithologies used and partial melting computation are described elsewhere^74^.

A symmetrical convergence velocity between the two continents is prescribed to facilitate subduction initiation with variable rates of 1, 3 and 5 cm per year, spanning the range of convergence rates in nature^75,76^ used in former experimental work^21^. The initial velocity is deactivated once the leading edge of the subducting slab reaches a depth of about 500 km, after which collision is driven exclusively by the pull of the subducting slab. The effect of the thermal age of the oceanic crust is explored by a variable oceanic age of 40, 60 and 90 Myr and an also variable oceanic length of 400 and 675 km. Reference models representative of the parameter field were further used to test the effects of using a stronger and weaker upper crust and a thinner thermal lithosphere in an additional 12 numerical experiments. The initial set-up can be found in the Extended Data Fig. 1 and the used parameters together with the results in Supplementary Table 1.

### Erosion and sedimentation model

Surface processes atop the crust are implemented as internal free erosion/sedimentation surface using the ‘sticky’ air approach. The surface of the lithosphere evolves according to the following Eulerian transport equation:

$$\frac{\partial {y}_{\mathrm{es}}}{\partial t}={v}_{y}-{v}_{x}\frac{\partial {y}_{\mathrm{es}}}{\partial x}-{v}_{{\rm{s}}}+{v}_{{\rm{e}}}$$

where *y*_es_ is a function of the horizontal distance *x* and defines the vertical position of the surface, *v*_*y*_ and *v*_*x*_ are the vertical and horizontal components of the material velocity field at the surface, *v*_s_ is the sedimentation rate and *v*_e_ is the erosion rate. The sedimentation and erosion rates are used for air and sea water, *v*_s_ = 0 mm yr^−1^ and *v*_e_ = 0.3 mm yr^−1^ when *y* < 9 km and *v*_s_ = 0.03 mm yr^−1^ and *v*_e_ = 0 mm yr^−1^ when *y* > 9 km.

### Density model

We use the extended Boussiniesq approximation with the incompressible continuity equation and variable density in the momentum and energy conservation equations. The density of rocks varies with temperature (*T*) and pressure (*P*) according to the relation

$${\rho }_{T,P}={\rho }_{0}\left[1-\alpha \left(T-{T}_{0}\right)\right]\left[\left(1+\beta \left(P-{P}_{0}\right)\right)\right]$$

In this expression *ρ*_*0*_ is the standard density at *T*_0_ = 298 K and *P*_0_ = 1 MPa, *α* = 2 × 10^−5 ^K is the coefficient of thermal expansion and *β* = 4.5 × 10^−12 ^Pa^−1^ is the coefficient of thermal compressibility.

Density plays a key role in determining the buoyancy of subducted materials and is influenced by phase transformations during gradual *P*–*T* increase. Our numerical models compute the transformations of olivine into wadsleyite and ringwoodite^77^, and into bridgmanite in the mantle^78^, and stishovite and perovskite in the crust^78^. Density changes associated with eclogitization of subducted crust are computed by linearly increasing the density of the crust with pressure from 0% up to 16% in the *P*–*T* region between the garnet-in and plagioclase-out phase transitions in basalt^79^. The physical parameters are described in Supplementary Table 1.

### Viscoplastic rheological model

The viscous and brittle (plastic) properties (Supplementary Table 1) are computed by evaluating the effective viscosity of the material. For the ductile rheology, the contributions from different flow laws are taken into account as described in the following relation:

$$\frac{1}{{\eta }_{\mathrm{ductile}}}=\frac{1}{{\eta }_{\mathrm{diff}}}+\frac{1}{{\eta }_{\mathrm{disl}}}$$

where *η*_ductile_ is the composite rheology after addition of the effective viscosities for *η*_diff_, diffusion creep and *η*_disl_, dislocation creep. The values for *η*_diff_ and *η*_disl_ are calculated after grain size evaluation. For the crust, a constant grain size is assumed, whereas for the mantle, grain size reduction and growth are effectively considered, with its numerical implementation being described elsewhere^40^.

The ductile rheology is combined with a brittle (plastic) rheology using the following upper limit for ductile viscosity:

$${\eta }_{\mathrm{ductile}}\le \frac{C+{\rm{\mu }}P}{2{\dot{\varepsilon }}_{{II}}}$$

$${{\mu }}={{{\mu }}}_{0}-{\gamma {{\mu }}}_{\gamma }\,\mathrm{for}\,\gamma \le {\gamma }_{0}\,{\mathrm{and}}\,{{\mu }}={{{\mu }}}_{1}\,\mathrm{for}\,\gamma > {\gamma }_{0}$$

$$\gamma =\int \sqrt{\frac{1}{2}{\left({\dot{\varepsilon }}_{{ij}\left(\mathrm{plastic}\right)}\right)}^{2}{\mathrm{d}}{t}}$$

In these relations *µ* is the internal friction coefficient, (*μ*_0_ and *μ*_1_ are respectively the initial and final internal friction coefficient; Supplementary Table 1), *μ*_*γ*_ = (*μ*_0_−*μ*_1_)/*γ*_0_ is the rate of faults weakening with integrated plastic strain *γ* (γ_0_ is the upper strain limit for the fracture-related weakening), *C* is the rock compressive strength at *P* = 0 (Supplementary Table 1), *t* is time and ${\dot{\varepsilon }}_{{ij}\left(\mathrm{plastic}\right)}$ is the plastic strain tensor. It is also assumed that the mantle inside outer-rise normal faults that reached the upper strain limit (*γ*_0_) is serpentinized and has the respective rheology (Supplementary Table 1).

### Model limitations

Our 2D models neglect along-strike variations, including complex 3D mantle flow effects, oblique plate geometries and the along-strike variations of collision or break-off. More importantly, our thermomechanical code employs simplified melting relations for independent lithologies and does not resolve hybrid melting between crustal and mantle components. Addressing this limitation motivates our integrated numerical–experimental approach: numerical models are used to constrain the physical conditions for mantle metasomatism, whereas experimental petrology tests whether these conditions produce geochemically meaningful melts. Our modelling reproduces first-order natural observations, including the temporal lag between collision and post-collisional magmatism and the composition of the magmatic products. At the same time, they highlight the need for future numerical routines that incorporate hybrid melting relations to more realistically model magmatism in subduction and collision settings.

### High-pressure experimental approach

Experiments were conducted in a Boyd–England-type piston cylinder apparatus in the Museo Nacional de Ciencias Naturales (Madrid, Spain) using half inch, talc-Pyrex assemblies. The starting material was assembled by mixing 8:2 and 1:1 proportions of a peridotite and representative compositions of an igneous or a sedimentary upper crust, simulating the interaction between the relaminated crust and the peridotite. The igneous composition is a granodioritic enclave from southwest Iberia^2^ that closely resembles the average composition of the upper continental crust^60^, whereas the sedimentary composition is a representative schist–greywacke melange from the Schist–Greywacke Complex in Iberia (Supplementary Table 2). As the occurrence of post-collisional magmatism long after ocean closure can limit external fluid availability, our experiments were conducted under fluid-absent conditions, utilizing only the structural water naturally hosted within the crustal lithologies (that is, hydrous minerals in the relaminated crust). Full homogenization of the starting material aimed to simulate focused flow interaction in the mantle^80^ after pervasive grain size reduction during collision, enhancing the reaction between the two components. Iron loss was prevented through the use of Pd_10_Au_90_ capsules and by keeping the experimental runs short in duration. Capsules were sealed by pressure welding and introduced into a MgO (crushable magnesia) pressure container. Temperatures were measured and controlled using Pt_100_-Pt_87_Rh_13_ thermocouples wired to Eurotherm EPC2000 controllers. The capsule was protected from the thermocouple using a thin MgO disc. Runs were brought to 1,200 °C and 1.5 GPa, held for 6–10 hours and then quenched by turning off the power. Experimental conditions were chosen to account for uncertainties in natural volatile contents and the potential for adiabatic lithospheric melting following mantle advection. Although our models predict near-solidus reactions in the mantle wedge, we used higher temperatures to generate sufficiently large glass pools for reliable trace-element analysis of the synthesized melts, a strategy previously used in experimental work^52^.

After quenching, the samples were sectioned, polished and metalized with graphite. Analyses of major elements of mineral phases and glasses of experimental capsules were performed with a JEOL JXA–8200 Superprobe equipped with four spectrometer WDS at the Universidad de Huelva. Glass major element measurements were performed with electron microprobe on large pools (>10–15 µm) to avoid contamination by nearby phases, except for runs containing large amounts of peridotite, where small glass pools required the use of a smaller beam diameter. Sodium and potassium migration was corrected using difference with the total through mass balance because the only phases are olivine and pyroxene and thus all Na_2_O and K_2_O must be in the liquid. Operating conditions were 15 kV for accelerating voltage and 20 nA for beam current. A combination of silicates, oxides and pure metals were used for calibration and ZAF correction procedures (wollastonite for Ca and Si, jadeite for Na, orthoclase for K, corundum for Al, periclase for Mg, metallic Fe and Ti for Fe and Ti). A defocused beam with 10 µm diameter was used for glass analyses to minimize Na migration. Analyses of trace elements were performed through 193 nm RESOlution SE laser system combined with an iCAP-Qc quadrupole ICP-MS in the Universidad del País Vasco. The NIST 612 glass was used for calibration^81^. Embedded and polished capsules were loaded with the external ablation cell and put on the stage of a reflected light petrographic microscope, allowing perfect positioning of the laser beam and selection of the appropriate spot size. Glass trace-element compositions were obtained in large pools using small spot sizes (20–25 µm). Reliability of analyses was controlled by repeated analysis of the international rock standards BCR-2G and GSD-1G^82^, obtaining a precision better than 10%. Two standard deviation uncertainties on shot-to-shot reproducibility are typically 2–5% on silicate phases unless signals are close to the respective detection limits, where counting statistics uncertainties may increase the overall uncertainty up to a few tens of percent.

### Attainment of equilibrium

Synthesized products consist of homogeneous glass and mineral assemblages. Glasses are featured as melt pools and along grain boundaries, with measured compositions displaying standard deviations lower than unity. A higher proportion of felsic contaminant effectively increases both the melt modal proportion and its silica content. Solid phases are mostly constituted by variable proportions of orthopyroxene, clinopyroxene and olivine. Phases are well crystallized and show euhedral faces against the glass pools. Run D131124 is the only exception, showing zonation in the synthesized phases but notably homogeneous liquid compositions, suggesting that the melt was in equilibrium with the available crystal faces. Modal proportions were obtained using a multi-variable least square calculation, resulting in residual values below 1%. In the case of FeO_t_, this value suggest that iron loss was successfully mitigated through the selected methods. All these considerations together suggest that equilibrium was successfully attained in the experimental runs.

## Online content

Any methods, additional references, Nature Portfolio reporting summaries, source data, extended data, supplementary information, acknowledgements, peer review information; details of author contributions and competing interests; and statements of data and code availability are available at 10.1038/s41561-026-01963-w.

## Supplementary information

Supplementary Table 1 Rheological properties used in the numerical experiments and numerical results.

Supplementary Table 2 Analyses of the starting materials and results of the high-pressure experiments.

Supplementary Table 3 Global geochemical compilation from post-collisional magmas.

Supplementary Video 1 Lithology evolution of model 13.

## Data Availability

All the relevant data and model outputs presented in this study are available via Zenodo at 10.5281/zenodo.19127108 (ref. ^71^) and Supplementary Information.

## Code availability

The numerical code I2ELVIS used for numerical experiments and the visualization scripts are available via Zenodo at 10.5281/zenodo.19127108 (ref. ^71^).
